# Impact of Physical Activity and Natural Bioactive Compounds on Endothelial Dysfunction in Chronic Kidney Disease

**DOI:** 10.3390/life11080841

**Published:** 2021-08-17

**Authors:** Elisa Grazioli, Annalisa Romani, Giulia Marrone, Manuela Di Lauro, Claudia Cerulli, Silvia Urciuoli, Arianna Murri, Cristina Guerriero, Eliana Tranchita, Manfredi Tesauro, Attilio Parisi, Nicola Di Daniele, Annalisa Noce

**Affiliations:** 1 Department of Exercise, Human and Health Sciences, Foro Italico University of Rome, 00135 Rome, Italy; elisa.grazioli@uniroma4.it (E.G.); claudia.cerulli@uniroma4.it (C.C.); ariannamurri@hotmail.it (A.M.); eliana.tranchita@gmail.com (E.T.); attilio.parisi@uniroma4.it (A.P.); 2 PHYTOLAB (Pharmaceutical, Cosmetic, Food Supplement, Technology and Analysis), DiSIA, University of Florence, Via Ugo Schiff 6, 50019 Sesto Fiorentino, Florence, Italy; annalisa.romani@unifi.it (A.R.); silvia.urciuoli@gmail.com (S.U.); 3 UOC of Internal Medicine—Center of Hypertension and Nephrology Unit, Department of Systems Medicine, University of Rome Tor Vergata, Via Montpellier 1, 00133 Rome, Italy; giul.marr@gmail.com (G.M.); dilauromanuela@gmail.com (M.D.L.); cristinaguerriero@hotmail.it (C.G.); didaniele@med.uniroma2.it (N.D.D.)

**Keywords:** cardiovascular disease, chronic kidney disease, endothelial dysfunction, endothelium, natural bioactive compounds, asymmetrical dimethylarginine

## Abstract

Chronic kidney disease (CKD) represents a world-wide public health problem. Inflammation, endothelial dysfunction (ED) and vascular calcifications are clinical features of CKD patients that increase cardiovascular (CV) mortality. CKD-related CV disease pathogenic mechanisms are not only associated with traditional factors such as arterial hypertension and dyslipidemia, but also with ED, oxidative stress and low-grade inflammation. The typical comorbidities of CKD contribute to reduce the performance and the levels of the physical activity in nephropathic patients compared to healthy subjects. Currently, the effective role of physical activity on ED is still debated, but the available few literature data suggest its positive contribution. Another possible adjuvant treatment of ED in CKD patients is represented by natural bioactive compounds (NBCs). Among these, minor polar compounds of extra virgin olive oil (hydroxytyrosol, tyrosol and oleocanthal), polyphenols, and vitamin D seem to exert a beneficial role on ED in CKD patients. The objective of the review is to evaluate the effectiveness of physical exercise protocols and/or NBCs on ED in CKD patients.

## 1. Introduction

Chronic kidney disease (CKD) is an increasing health problem both socially and economically, worldwide [1]. Patients in renal replacement therapy (RRT) have high mortality, mainly related to cardiovascular diseases (CVDs) [2,3]. The enhanced incidence of cardiovascular (CV) events is closely related to the state of chronic inflammation, typical of CKD, resulting in the acceleration of ageing phenomena. In fact, CKD is currently considered to be an early ageing model [4].

**Figure 1:**
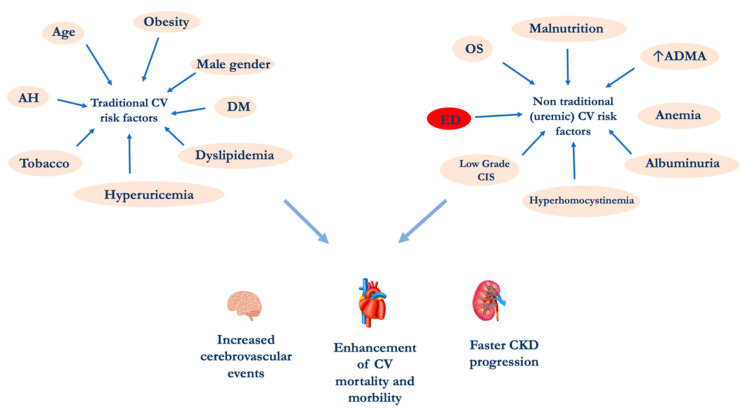
CV risk factors in CKD patients. Abbreviations: AH, Arterial Hypertension; ADMA Asymmetric dimethylarginine; CIS, chronic inflammatory state; CKD, chronic kidney disease; CV, cardiovascular; DM diabetes mellitus; ED, endothelial dysfunction; OS, oxidative stress.

An important epidemiological study demonstrated that hemodialysis (HD) patients aged 25–35 years have a higher CV-mortality rate than subjects over 85 years of the general population. This phenomenon cannot be explained by examining the traditional “modifiable” and the “non modifiable” CV risk factors such as gender, age, tobacco habit, etc. [5,6,7,8]. For this reason, CV risk factors related to uremia need to be considered. These specific CV risk factors are multiple: (i) alterations of calcium-phosphorus metabolism, (ii) hyperhomocysteinemia, (iii) endothelial dysfunction (ED), (iv) oxidative stress (OS), (v) chronic inflammation, (vi) increased asymmetric dimethylarginine (ADMA), (vii) albuminuria, (viii) malnutrition and (ix) uremic sarcopenia (Figure 1) [9,10]. Among these, ED plays a pivotal role in the increase CV morbidity and mortality. The endothelium, previously considered a barrier selectively permeable between the blood and the vascular wall, is today recognized as a crucial organ in the regulation of the vascular tone and the structure [11]. Endothelial cells represent a complex and dynamic system able to respond to stimuli of different nature, having a large number and type of receptors and the capacity to produce a series of substances able to act on different pathways.

Some authors have highlighted sexual dimorphism in endothelial cells [12,13]. These macro and micro vascular differences appear to be related to the protein synthesis by endothelial cells. In particular, the differences between the two sexes are: (i) the increased expression of androgen receptors, of estrogenic receptor (ER)-α and of vascular adhesion molecule-1 (VCAM-1) in men compared to women, (ii) the enhanced expression of platelet endothelial cell adhesion molecule-1 (PECAM-1), integrin α_v_β_3_, intracellular adhesion molecule-1 (ICAM-1) and neural cadherin (N-CAD) in females, while ER-β and VE-cadherin (VE-CAD) do not show differences between the two sexes [14].

Physiological endothelial functions include the production of adhesion molecules, the platelet activation and the production of factors involved in the coagulation cascade and in the fibrinolytic system, the regulation of inflammatory response, the cell proliferation, and the control of the vascular tone. Endothelial cells constitute a real endocrine-autocrine-paracrine organ [15,16]. The endothelium, therefore, exerts a significant role in the process of angiogenesis, in the vessel permeability, in the regulation of platelet activation and in the inflammation [17,18].

Recent studies have underlined the role that physical exercise and natural bioactive compounds (NBCs) may have separately in the prevention of ED in CKD patients [19,20,21,22]. Nevertheless, nowadays there are few studies in the literature that investigate the effects induced by the physical exercise associated with NBCs assumption in CKD patients [23,24,25,26].

Some authors demonstrated how NBCs (such as curcumin) and the regular practice of aerobic exercise significantly improve endothelial function [23]. Akazawa et al. compared the assumption of 150 mg of curcumin *per* day for 8 weeks with the practice of aerobic exercise training more than 3 days *per* week for 8 weeks (2–3 supervised sessions and additional home-based training) on vascular endothelial function. The authors divided the study population in three subgroups: (i) curcumin group, (ii) exercise group and (iii) control group. Flow-mediated dilation increased significantly and equally in the curcumin and exercise groups, while no changes were detected in the control group. In this study, the authors evaluated separately the effects of the two interventions, but it may be interesting to understand how the combined effects could impact on ED.

A recent study [24] investigated the role that curcumin may have on kidney disease when it is associated with aerobic exercise. Authors examined the action of curcumin in a CKD animal model that practiced physical exercise. They demonstrated that together the curcumin and aerobic exercise improved the kidneys histopathology, and this observed healthy effect does not occur only with the physical activity. In this way, the combination of physical exercise and curcumin could induce a nephroprotection, acting in a synergic manner.

In another study, Zanuzo et al. investigated the role of chronic vitamin D supplementation associated with a regular aerobic exercise (like swimming) on kidney injuries. Authors conclude that physical exercise associated with vitamin D supplementation can prevent renal injury. Unfortunately, this study was conducted on rats, but it may be interesting to evaluate the same effects on humans [25].

Finally, Kutlu et al. demonstrate in rat model that daily moderate exercise associated with vitamins C and E supplementation could exert a beneficial effect thanks to their anti-oxidant action and could help to prevent the development of diabetic nephropathy in diabetic animal models [26].

Due to these evidences, the aims of our review will be to evaluate: (i) the correlation between ED and CKD; (ii) the possible beneficial and protective effects mediated by adapted physical activity and NBCs, taken individually or in combination, on ED in CKD patients.

## 2. Chronic Kidney Disease and Endothelial Dysfunction

Chronic nephropathy is a systemic disease that currently affects about 10% of the general population [27]. The CKD increased prevalence is certainly due to a global ageing of the population and especially to the increase in the incidence of metabolic diseases such as diabetes mellitus (DM), metabolic syndrome (MetS), obesity, etc. [28].

The association between CKD and CVD was first highlighted in end-stage renal diseases (ESRD) patients in dialytic treatment [2]. More recently, it has been shown that even the presence of slight changes in renal function, such as albuminuria, leads to a significant enhancement in CV events, which are more frequent compared to the progression versus ESRD.

CKD causes ED through several mechanisms. Physiologically, the vascular endothelium can be considered a “real organ”, able to secrete, in response to a great variety of signals, numerous chemical mediators [29,30]. Among the main physiological functions of the endothelium, the nitric oxide (NO) release represents a milestone. This molecule displays several functions in the human body such as the regulation of neuronal communication, inflammatory and immune responses, and the regulation of vascular tone [31,32].

ED represents an abnormality that develops at the level of the tunic that covers the internal surface of arterial and venous vessels and more precisely an alteration of normal endothelium, which results in the loss of some structural and/or functional characteristics [33,34].

In particular, at renal level it is possible to distinguish different types of endothelial cells, such as those of the small vessels that regulate the blood flow in the kidney and are also involved in the coagulation process and in the regulation of vascular permeability and in the inflammatory state. The glomerular endothelial cells, in addition to the already mentioned functions, are involved in glomerular filtration and in providing support to the podocytes. Finally, the endothelial cells of the microvasculature in the kidney tubules participate in the tubular reabsorption [35,36]. As for the kidney dysfunction, it has been highlighted a transition of kidney endothelial cells into mesenchymal phenotype, phenomenon called endothelial-to-mesenchymal transition, promoting renal fibrosis. Therefore, in CKD patients the accumulation of uremic toxins worsens the residual renal function and induces the systemic ED, contributing to cause CV comorbidities [37]. In fact, the increased incidence of CV events in CKD patients is related to the uremic toxins but also to the chronic inflammation state, typical of this pathological condition [38]. The evidence of a close relationship between kidney and hearth lays the foundations for a wider and more detailed stratification of the CV risk and suggests the opportunity for a correct and prompt management of CV comorbidities, starting from the early stages of CKD [39].

During CKD, the ED development appears to be related to a decrease in the inducible nitric oxide synthase (iNOS) activity and an increase in ADMA levels [31]. The latter gradually increases with the decline of the renal function. In fact, ADMA concentration is related to CKD progression, and it is an important CV risk factor both in the general population and in nephropathic patients [40]. ADMA is synthetized endogenously by the degradation of methylated proteins [41]. The asymmetrical residues of methylated arginine such as ADMA itself and NG-monomethyl-L-arginine (L-NMMA), inhibit the NO synthesis, as they are competitive inhibitors of NO synthase (NOS). ADMA and L-NMMA are excreted in the urine by renal pathways, and in CKD patients it can be observed an enhancement ADMA levels [42]. The ADMA accumulation in nephropathic patients could be related to renal parenchyma damage, resulting in a reduced expression and activity of dimethylarginine dimethylaminohydrolase (DDAH) [43]. This enzyme is involved in ADMA metabolism, in fact DDAH induces the ADMA hydrolytic degradation into L-citrulline and dimethylamine [44]. In some pathological conditions, such as DM, hyperhomocysteinemia, OS and dyslipidemia, DDAH reduced levels have been observed [45]. A decreased activity of DDAH induces an increase in ADMA concentration and subsequently a decreased NO production in the endothelium [46]. For this reason, ADMA has been described as a compound able to induce ED. In fact, intra-arterial local infusion of ADMA seems significantly reduce the blood flow in forearm [47], while the intravenous one causes an enhancement of blood pressure values of 6% and of systemic vascular resistance of 24% [48]. Another cause of ADMA accumulation in CKD patients, it is represented by an increased rate of protein turnover that, in turn, causes enhanced concentrations of methylated protein [49]. A study by Damaso et al. [50] conducted on a cohort of 225 HD patients, highlighted also an interaction between inflammation biomarkers and ADMA. In particular, the mortality was higher in the group that had both elevated ADMA and C-reactive protein (CRP) levels compared to groups that had only one of the biomarkers altered. The authors concluded that inflammation amplifies the risk of death and CV events in HD patients with ADMA high levels. Therefore, further studies are needed to establish normal serum and urinary ranges of ADMA for different ages. The link between ED, increased ADMA levels and OS is represented by the impaired NO production. In fact, NO, as above mentioned, performs several biological functions [51]. The reaction catalyzed by NOS enzyme, starting from L-arginine, produces NO and L-citrulline [52,53,54]. In blood vessels, endothelial NOS (eNOS) represent the most abundant isozyme. Under pathological conditions, eNOS can produce reactive oxygen species (ROS) [55]. In turn, the OS can induce an alteration in NO production through two mechanisms involving eNOS enzyme: the first one is represented by the eNOS inhibition and the second one is due to uncoupling eNOS. Consequently, the decreased NO production contributes to cause ED [56]. Among the factors related to OS during CKD, it should be considered the accumulation of uremic toxins such as indoxyl sulfate (IS), homocysteine (Hcy), and advanced glycation end products (AGEs) [57,58].

**Figure 2:**
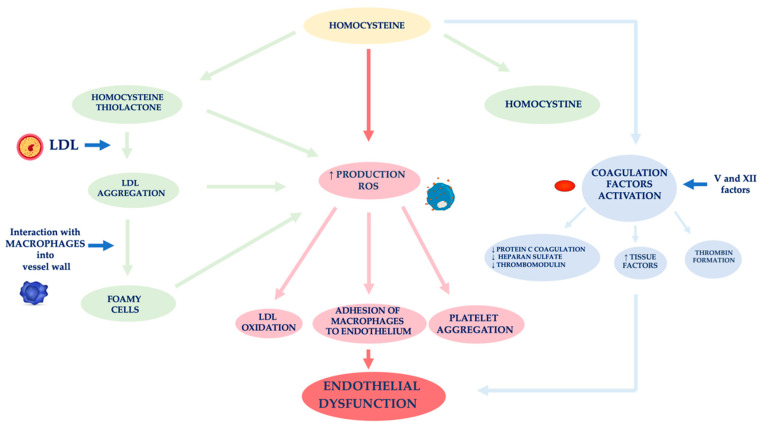
Mechanisms of vascular wall damage induced by homocysteine. Abbreviations: LDL, low-density lipoprotein; ROS, reactive oxygen species.

In nephropathic patients, another factor related to ED is hyperhomocysteinemia (Figure 2) [59]. Hcy is an amino acid sulfide, placed at the crossroad of a complex metabolic pathway. Normal Hcy levels are between 5 and 15 μmol/L [60], while hyperhomocysteinemia is defined when Hcy concentration is above 15 μmol/L [61].

Factors affecting an increase of Hcy plasma levels can be genetic and/or acquired. As for the former, several mutations have been identified in the various genes involved in Hcy metabolism. The most analyzed mutation is certainly that relating to the methylenetetrahydrofolate reductase (MTHFR) gene. The acquired factors include gender, age, kidney function and lifestyle [62]. In fact, Hcy levels in CKD patients are on average increased because of its reduced kidney metabolism, namely between 20 and 40 μmol/L, especially if it is also present a folate and B12 vitamin deficiency, malnutrition, accumulation of uremic toxins [63,64,65,66]. Several authors demonstrated that Hcy plasma concentration is directly related to plasma creatinine values as it has been shown that in the renal parenchyma there are enzymes involved in Hcy remethylation and transsulfuration [67,68,69]. Jansen et al. hypothesized that hyperhomocyteinemia in CKD patients is mainly related to the slowing of its catabolic pathway of transsulfuration [70]. In fact, the enzymes cystathionine-β-synthase and cystathionine-γ-lyase are located in the proximal tubules of the nephron. However, the Hcy excretion in the urine is limited to 0.1% of the Hcy total, as the latter is largely reabsorbed at the tubular level. Unlike cystine, which has an antioxidant action, Hcy has a pro-oxidant action, as it promotes the formation of free radicals. The different behavior of these two amino acids is related to both an Hcy auto-oxidation and its enzymatic transformation into thiolactone, a substance with a strong oxidizing power [71]. The formation of thiolactone induces structural changes in intracellular proteins up to the loss of the biological activity of native proteins. The thiolation process can also occur at level of extracellular components, such as apolipoprotein B (apoB) and low-density lipoprotein (LDL). Hcy forms, with the LDL, aggregates which are captured by macrophages in the vessel wall. These phagocytic cells are transformed into foamy cells that release other free radicals that induce the oxidation of LDL, the platelet aggregation and the adhesion of macrophages to the endothelium [72,73,74]. Meanwhile, the Hcy autoxidation induces the production of further free radicals (hydroxyl) that trigger a process of lipid peroxidation at the level of the endothelial membranes [75,76].

ED induced by Hcy is also caused by the activation of coagulation factors, such as V and XII, whose stimulation induces: (i) a reduced expression of the coagulation protein C, of the thrombomodulin and of the heparan sulfate by the endothelium, (ii) an increased expression of the tissue factors and the thrombin formation [77]. In physiological conditions, the endothelium develops defensive mechanisms against the toxic action induced by hyperhomocysteinemia, releasing NO which forms S-nitrous-homocysteine, a compound that inhibits the production of hydrogen peroxide. S-nitrous-homocysteine has an important vasodilating action and inhibits platelet aggregation [78]. However, the persistence of the damage produced by a chronic condition of hyperhomocysteinemia, progressively reduces the ability of the endothelium to produce NO. An in vitro study by Tyagi et al. showed that Hcy activates the protease-activated receptors 4 which induces the production of ROS through an increase in NADPH oxidase and a decrease in expression of thioredoxin. Hyperhomocysteinemia also reduces the bioavailability of NO through the increase in NO_2_-tyrosine and through the accumulation of ADMA caused by the reduced expression of DDAH [79].

Furthermore, Hcy appears to stimulate the release of interleukin (IL)-8 and macrophage chemotactic factor (MCF). These chemokines have specific chemotactic activity for monocytes and neutrophils. The infiltration of the arterial wall by monocytes is a key event for the induction of atherogenesis. Instead, the monocyte chemoattractant protein (MCP) stimulates the migration of monocytes into the intima of the vessel wall. The OS induced by Hcy, on the one hand, directly damages endothelial cells, and on the other hand, induces the expression of matrix metalloproteases (MMPs), enzymes responsible for remodeling the vessel wall. In physiological conditions, MMPs are in equilibrium with their inhibitors, while in pathological conditions, this equilibrium is unbalanced towards a decrease in MMPs contextual to the increase in their inhibitors [80,81].

Another mechanism causing ED in CKD is an unbalance of the calcium-phosphorus metabolism. In particular, phosphate retention induces the development of CKD-mineral bone disorder (CKD-MBD) which, in turn, contributes to cause vascular calcifications. In fact, numerous epidemiological studies have shown that higher serum phosphorus levels, even in the absence of CKD, represent a risk factor for CVDs [82,83]. During CKD, vascular calcifications of the intima and media are frequent and are correlated to vascular rigidity. Elevated phosphate levels trigger the transformation of smooth muscle cells of the arterial wall into an osteoblast-like phenotype [84,85]. In addition, hyperphosphatemia affects ED, increasing apoptosis, inducing an increased production of ROS, impairing the NO production and decreasing the expression of annexin II [86,87]. The latter is a glycoprotein involved in various cellular functions, including the motility of epithelial cells, the fibrinolysis, the formation of anion channels and the interaction with matrix cells [88].

## 3. Search Methods

**Figure 3:**
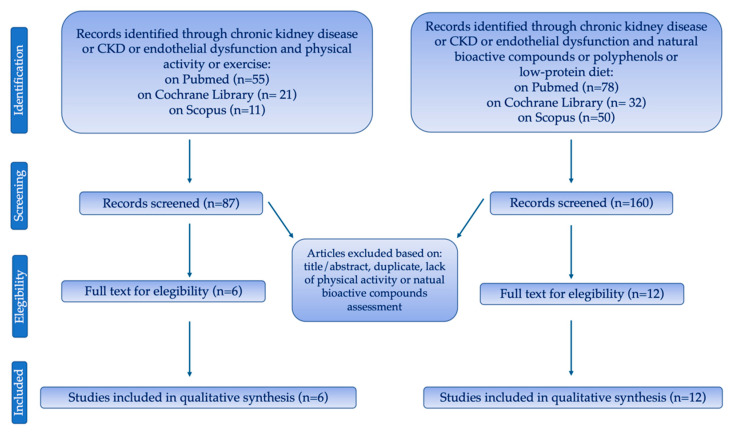
Flow-chart of search methods.

The purpose of this article is to review a series of studies evidencing the possible positive and protective role of adapted physical activity and NBCs in CKD patients, alone or in combination, on ED. A literature search was conducted using three databases (PubMed, Scopus and Cochrane Library). The search was limited to peer-reviewed journals written in the English language and the search terms were “chronic kidney disease” in combination with “physical activity” AND “exercise” AND “endothelial dysfunction” AND “endothelial function” AND “aerobic training” AND “resistance training” AND “heart rate” AND “NBCs” AND “polyphenols” AND “hyperhomocysteinemia” AND “low-protein diet”. The full search was manually retrieved (Figure 3).

## 4. Physical Exercise and Endothelial Dysfunction

The typical comorbidities of CKD, such as OS, chronic low-grade inflammation, metabolic acidosis, reno-parenchymal hypertension, uremic sarcopenia, CKD-MBD, insulin resistance, contribute to reduce the performance and the levels of the physical activity in nephropathic patients [89,90]. So, physical capacity of CKD patients is lower than healthy subjects, and it decreases quickly after the beginning of HD, leading to the loss of functional independence and to the decrease in the quality of life parameters [91].

Downey et al. [92] demonstrated that CKD patients had a lower brachial artery flow-mediated dilation that correlates with an augmented blood pressure (BP) response during exercise and a lower peak oxygen uptake (VO_2_peak) [92]. So, the authors suggest that ED that characterizes CKD patients may induce a higher BP response to physical exercise and it may explain the scarce exercise tolerance observed in this population [92]. At the same time, Sprick et al. underline that CKD patients show relevant increase in BP during exercise, and this condition is associated with an augmented risk of CV mortality [93,94]. Interestingly, they advise that this amplified BP response may be associated with ED. The authors evaluated the effect of 12 weeks of aerobic exercise training (cycling exercise 45 min-session, three times *per* week) on BP and endothelial function (assessed via peripheral artery tonometry) during maximal exercise in CKD patients. They compared the results with a group of patients who practiced stretching protocol for the same period. At the end of the training period, the authors showed that VO_2_peak and reactive hyperemia index increased in the aerobic exercise group, but not in the stretching group. Moreover, a slight decrease in systolic blood pressure (SBP) from pre- to post-intervention was observed in the aerobic exercise group. These data suggested that 12-weeks of structured aerobic exercise training could attenuate BP responses during maximal exercise and improve endothelial function in CKD patients [93].

Even though these results seem promising, physical activity is not always recommended in CKD patients because it could impair renal residual function and increase proteinuria [95]. However, recent studies underline the important preventive role that physical exercise may have in CKD patients both for renal and for cardiovascular systems. Both aerobic and resistance training (RT) have been proposed as possible treatments to reduce obesity, inflammation, ED, OS, insulin resistance, and CKD progression. The effects of aerobic training seem to be different: in non-dialysis CKD patients, this type of training contribute to decrease microalbuminuria, helps to protect from OS, and may increase the glomerular filtration rate (GFR). In HD patients, aerobic training has been reported to enhance insulin sensitivity, to increase hemoglobin and strength, to decrease BP, to ameliorate lipid profile, and to improve quality of life. On the other hand, RT in the non-dialysis CKD population has been reported to reduce inflammation, to increase serum albumin, to maintain body weight (BW), to increase muscle strength, insulin-like growth factor-1 (IGF-1) and GFR. This type of exercise in HD patients seem to increase either muscle strength or physical functionality and to improve IGF-1 status [96].

Hiraki et al. underline how 30 min of combined physical activity (both aerobic and RT) performed three times *per* week can increase strength in upper and lower limbs without alteration of estimated-GFR (e-GFR) or of proteinuria [97]. At the same time, Baria et al. highlight how aerobic exercise at low-moderate intensity (40–60% maximal oxygen uptake-VO_2_max) practiced three times a week, 30 min *per* session, can decrease visceral fat and waist circumference and increase lower limb strength in CKD patients [98].

Greenwood et al. [99] evaluated the effect of moderate-intensity combined exercise training on kidney function in CKD patients with progression of disease from stage III to IV. They examined the effect of resistance and aerobic training (3 days *per* week) performed for 12 months in a group of CKD patients compared to a control group that received standard care. Specifically, they evaluate eGFR, pulse wave velocity (PWV), VO_2_peak and waist circumference. After 12 months of training, it was observed a significant mean difference between exercise group and control group in rate of change in eGFR; the exercise group demonstrated a slower decline in eGFR. Significant between-group mean differences were observed in PWV, waist circumference and VO_2_peak, revealing a general improvement of all these parameters in the exercise group. Despite the results indicate a positive effect of the physical exercise, this study was conducted on a small group of patients (only 20 subjects). To reach a major result on the effect of physical exercise training on kidney function, it should be necessary to investigate a larger number of CKD patients and with a longer follow-up period [99]. However, studies demonstrate that aerobic exercise represents a valid tool to improve ED in CVDs [100,101,102,103] and a new evidence from animal studies suggests that exercise may have similar positive effects on ED in CKD [104,105,106].

In recent years, many efforts have been made to understand how physical exercise can play a role in improving endothelial function for reducing the burden of CVD in CKD patients [107].

The latest evidence from Martens et al. suggests the hypothesis that aerobic exercise training could improve endothelial function in CKD by reversing impairments to the L-arginine transport system, thus facilitating substrate delivery for NO production [108]. Since ED becomes more difficult to reverse in advanced CKD, aerobic exercise training may represent a novel adjunctive therapy in treating ED from the early stages of CKD.

Headley et al. were the first who examined the effect of moderate-intensity training on kidney and vascular function in CKD patients. Specifically, they proposed 48 weeks of moderate-intensity aerobic exercise training (50–60% VO_2_max), performed three times a week, lasting 55 min *per* session, and they evidenced a significant improvement in aerobic capacity of patients (VO_2_max), as well as the quality of life, without a worsening in kidney residual function after this training (Table 1) [19]. In another study, the same authors tried to analyze the effect of short-term moderate intensity exercise on arterial stiffness in stage III CKD patients. Arterial stiffness was assessed with aortic PWV and the aerobic capacity using the VO_2_peak. After 16 weeks of aerobic training at 50–60% VO_2_max, three times a week, 15–30 min *per* session, the VO_2_peak significantly increased in the exercise group of 1.6 mL/kg/min (or 8.2%), whereas the same value in the control group declined by 0.5 mL/kg/min (approximately 2.8%). Quality of life scores for physical functioning, vitality and bodily pain were higher in the treatment group compared to the control group. Although the PWV did not change significantly [109], the result on PWV could be explained by the low exercise intensity, since Hayashi et al. [110] reported a decrease in PWV value after a similar aerobic training at an intensity of 60–75% of the heart rate reserve, in 17 healthy sedentary middle-aged men (before exercise: 937 ± 34 m/s; after exercise: 871 ± 32 m/s).

Following these results, Kirkman et al. investigated the effect of moderate to vigorous aerobic exercise on vascular function in non-dialysis CKD patients. In this randomized controlled trial, 36 patients practiced 45 min of supervised exercise (cycling, walking/jogging, or elliptical) 3 days a week at 60–85% heart rate reserve for 12 consecutive weeks. Microvascular function was assessed via cutaneous vasodilation during local heating, measured by laser-Doppler flowmetry coupled with microdialysis; conduit artery function was assessed via brachial artery flow-mediated dilation; aortic pressure waveforms and PWV were acquired with tonometry and oscillometry. Results were compared to a control group which received routine care, without changes in their current standard care. The authors concluded that 12 weeks of moderate-vigorous aerobic exercise improved cardiorespiratory fitness, maintained conduit artery and endothelial function while these parameters declined in the control group. They also found evidence for an improved microvascular function in CKD patients involved in training, (87 ± 2% vs. 91 ± 2%) compared to the control group (86 ± 2% vs. 84 ± 3%), suggesting that exercise may have preventive implications for the development of CVD in CKD. Although aerobic exercise did not affect arterial stiffness, the authors underline that exercise-induced shear stress is provoked by 45 min exercise session. Lastly, brachial artery flow-mediated dilation was maintained after training (2.6 ± 0.4% vs. 3.8 ± 0.8%) while it decreased in the control group (3.5 ± 0.6% vs. 2.3 ± 0.4%) [20]. On the other hand, a different study [111] does not confirm the results reported above; Van Craenenbroeck et al. evaluated the effects of 3 months home-based aerobic training program consisted of four daily cycling sessions of 10 min (at 90% of the heart rate achieved at the anaerobic threshold). In particular, the authors evaluated endothelial function (with flow-mediated dilation of the brachial artery), aerobic capacity (VO_2_peak), and arterial stiffness (carotid-femoral PWV). The results showed an improvement only in the VO_2_peak analysis, probably because they proposed a non-supervised home-based exercise program, where patients performed four bouts of 10 min of exercise not consequently, but throughout the whole day, while Kirkman et al. prescribed supervised and continuous 45 min sessions [20]. No improvement was found in cellular markers for vascular function, evaluated through the numbers of endothelial and osteogenic progenitor cells.

Other studies were conducted to evaluate the role of physical exercise training in HD patients. These patients present increased inflammation, unbalanced redox profiles and elevated biomarkers related to ED. The study of Corrêa et al. was conducted to verify the effects of 3 months of RT on redox balance, NO bioavailability and inflammation profile, in HD patients. Fifty-five maintenance HD men were recruited and randomized into either a control or RT group. The RT program was structured in 50 min sessions, three sessions *per* week for 12 weeks while the patients were receiving dialysis (intradialytic exercise). RT repetitions balanced concentric and eccentric lifting phases were supervised by a strength and conditioning specialist [114]. The RT sessions consisted of different exercises: (i) chest press with Thera-band and rowing with Thera-band; (ii) dumbbell biceps curl; (iii) unilateral overhead triceps extension; (iv) unilateral shoulder abduction, and shoulder press with dumbbells; (v) bilateral knee extension with weights attached to the ankles; (vi)weight hip thrust; (vii) hamstrings curl with Thera-band; (viii) hip adduction with Thera-band; (ix) hip abduction with Thera-band and seated plantar flexion with fixed weights ranging from 2 to 15 kg. Upper body exercises were performed with the limb without the arteriovenous fistula. At the end of the training period (12 weeks), patients who practiced RT demonstrated an improvement both in redox and inflammatory profiles, where the thiobarbituric acid reactive substances and TNF-α decreased, while total antioxidant capacity and IL-10 increased. Moreover, the exercise group showed an improvement in biomarkers of endothelial function, reporting an increasing of NO_2_^−^ and a decreasing of ADMA. This group also improved muscle strength both in upper and lower limbs [112].

A recent study demonstrated that an intradialytic aerobic cycling exercise program is also possible in advanced CKD patients [115]. The training program consists of cycling for a 30 min session (5 min warm-up, 20 min of cycling at the desired workload, and a 5 min cool down) for three sessions *per* week for 3 months. Exercise was effective in reducing inflammatory markers, in particular patients reported a lower level of serum inflammatory cytokines, including IL-6 and high sensitivity (hs)-CRP. The training group also showed a statistically significant improvement in circulating CD133, CD34 and kinase insert domain-conjugating receptor-positive endothelial progenitor cells. Moreover, the aerobic cycling seems to increase cardiovascular endurance and functional capacity (6 min walking distance), and to attenuate the loss of femoral neck bone density in HD patients. It was well tolerated by all participants and there were no complications. So intradialytic aerobic cycling exercise may represent a safe and economic approach to reduce inflammation and improving CV endurance, bone density and functional capacity in HD patients [115].

Additionally, district training was evaluated to understand what kind of changes can determine on vessels. In particular, Rus et al. evaluated the influence of handgrip training and intermittent compression of the upper arm veins on forearm arteries and veins. The authors selected 18 HD patients who executed daily handgrip training using a rubber ring, together with daily intermittent compression of the upper arm veins by elastic band; the training lasted 8 weeks. The parameters that they evaluated were forearm circumference, maximal handgrip strength, arterial and vein parameters (the radial artery diameters, the endothelium-dependent vasodilation, the distensibility of veins). All these parameters were measured at the beginning of the study, and after 4 and 8 weeks (using ultrasound scanning). Results showed an increase in the radial artery diameters after training (before activity: 1.89 +/− 0.10 mm, after activity: 1.95 +/− 0.10 mm), and endothelium-dependent vasodilation after 4 and 8 weeks of activity. Moreover, after 8 weeks of training, the venous parameters before tourniquet placement improved (before activity: 2.40 +/− 0.16 mm, after activity: 2.62 +/− 0.17 mm), as well as the venous parameters after tourniquet placement (before: 3.36 +/− 0.17 mm, after 4 weeks: 3.51 +/− 0.18 mm, after 8weeks: 3.68 +/− 0.18 mm). At the end of the study, the authors concluded that handgrip training and intermittent compression of the upper arm veins, performed daily, increased the diameter of forearm arteries and veins and improve endothelium-dependent vasodilation [113].

As suggested in the literature, aerobic exercise training may represent a new tool to act on ED in CKD. The mechanism by which aerobic exercise training improves endothelial function is associated with an increase in endothelial NO bioavailability and with a reversing impairment to the L-arginine transport system, thus facilitating substrate delivery for NO production. Moreover, aerobic training determines an increase in blood flow that produces tangential and circumferential shear forces on the endothelial surface. Shear stress represents a stimulus for endothelial NO production, and it is the most important mechanism by which aerobic exercise improves endothelial function in CVDs [107]. The same shear stress determines an increase in L-Arginine levels, which represents a primary substrate for NO synthesis [105]. This pathway suggests that regular aerobic exercise training may be an effective therapy to contrast the impairments in L-arginine transport that occur with CKD. Inverting the reduction in endothelial L-arginine transport may also improve cardiac function, through increased NO production within coronary arteries and their consequent dilation [107]. Furthermore, the association between exercise and L-arginine transport may reach optimal results when physical exercise and L-arginine supplementation are used in combination. Recent studies demonstrate that the supplementation with L-arginine alone does not reverse ED in CKD rats, but when this supplementation was combined with physical exercise training, it could produce an increase in endothelial function in CKD [108]. The latest studies were conducted on rats, but we need more studies on humans to demonstrate the effect of exercise training on mediators of vascular disease. The only study conducted on humans that tried to investigate whether an aerobic exercise training program improves peripheral endothelial function in stages III-IV CKD patients, showed that exercise significantly improved VO_2_peak, but not vascular function [111]. So, further studies are necessary to better understand the real effect of physical exercise on ED in uremic patients.

## 5. Impact of Natural Bioactive Compounds on Endothelial Dysfunction

NBCs are present throughout the plant world and have different properties for the survival of the plant and for its defense. In the last decade the most studied class of NBCs is that of phenolic and polyphenolic compounds [116]. They are naturally present in plant tissues and are responsible for the color, aroma and flavor of many foods and they are made up of different subclasses such as: anthocyanins, flavanols, flavones, isoflavones catechins and proanthocyanins, condensed and hydrolysable tannins, hydroxycinnamic acids [117]. They represent an important part of the human diet and are present in all vegetables, cereals, legumes and fruit, and in some beverages such as red wine, beer and green tea. For decades, polyphenols have interested many researchers for their protective action against OS. The NBCs of Mediterranean diet (MD) exert a positive effect on CVD and in particular on ED. In the typical foods of MD, especially in olive fruit and in extra virgin olive oil (EVOO), there are bioactive molecules such as hydroxytyrosol, oleuropein aglycone, tyrosol, 10-hydroxyoleocanthal and oleocanthal [118]. The free and bound hydroxytyrosol is the most studied molecule as antioxidant for its high bioavailability. The European Food Safety Authority (EFSA) health claim is related to hydroxytyrosol content as it is able to confer cardiovascular protection [119,120].

The oleocanthal has not only antioxidant but also anti-inflammatory properties as described in 2005 [121].

In red fruit, such as strawberries, blueberries, raspberries and red grapes, there are bioactive molecules such as anthocyanins which have anti-radical and antioxidant activity. 

An interest study has been conducted on CKD patients, evaluating the possible beneficial effects of Italian Mediterranean Diet (IMD) in combination with Italian Mediterranean Organic Diet (IMOD) (Table 2). The NBCs present in IMOD and IMD are mainly represented by phenolic compounds with antioxidant and anti-radical activity. This study demonstrated that the IMOD was associated with significant and positive changes in body composition of nephropathic patients, despite no alteration in total energy intake (kcal/day), physical activity and lifestyle was observed during this study. The reduction in Hcy and of other CV risk factors such as total phosphate, serum total cholesterol and albuminuria, confirm the superior quality of the IMOD nutritional intervention. In fact, due to the high content of folate, vitamin B12 and polyphenols, IMOD induces a better metabolism of Hcy, counteracting hyperhomocysteinemia [63]. Moreover, the highest quality of IMOD seems to be related to a reduction in phosphate, factor primary involved in ED in nephropathic patients.

Folic acid has also shown to lower Hcy levels in several clinical conditions, and it can improve brachial artery endothelial function. In uremia, a relative resistance to folic acid is usually found, but its supplementation in CKD adult patients at doses comprised between 5 and 15 mg/day, can decrease Hcy levels from 40% to 50% compared to basal values. However, the impact of folic acid on endothelial function in CKD patients is still debated. One study shows that in CKD children, supplementation with high-dose folic acid for 8 weeks induces a reduction in Hcy levels, decreases LDL oxidation and improves endothelial function. These promising findings differ with the disappointing effects of folic acid supplementation on vascular function in CKD adult patients. It was selected the population composed by children to study the process of early atherosclerosis in its natural history. In addition, the young population offered an opportunity to minimize the incalculable impact of lifelong confounding risk factors on endothelial function [122]. However, long-term benefits of folic acid supplementation necessitate further studies.

Metabolic acidosis is another factor able to increase CV risk and it concurs to CKD progression, causing an increase in aldosterone, endothelin and angiotensin II, factors related to ED. Low-protein diet (LPD) seems to be a possible treatment able to counteract metabolic acidosis, but this nutritional approach if it is not well balanced, it could induce malnutrition [131].

Another possible NBC, useful for counteracting the ED in CKD patients, is the inulin, which should be supplemented in combination with LPD. Interestingly, a study conducted by Lai et al., demonstrated a significant reduction in CRP levels in patients who received LPD plus inulin and this reduction in the inflammatory state was also associated with a decrease in ED markers. This intervention was also correlated with the improvement in psycho-cognitive parameters that are very important for a better quality of life and the physical well-being of CKD patients [123].

Another possible treatment, based on NBCs, investigated the improving of endothelial function secondary to an increase in NO bioavailability caused by L-arginine. According to animal studies, L-arginine is able to attenuate the progression of atherosclerosis. This study examined whether dietary L-arginine supplementation improved endothelial function in CKD children. After oral L-arginine assumption, plasma L-arginine levels significantly increased, but no significant changes were observed in endothelial-dependent dilation during L-arginine or placebo administration [124].

Increases in OS have also been addressed as one potential cause for the quickened atherosclerosis of CKD patients. Ascorbate represents one of the most important antioxidants both plasmatic and intracellular, exerting beneficial effects by inhibiting lipid peroxidation and reducing ED. However, in the presence of transition metals such as iron, ascorbate may increase the generation of oxidant agents and ascorbylation may induce additional carbonyl stress to uremic patients. HD patients showed lower ascorbate plasma levels compared to healthy subjects, mostly due to its loss into the dialysate. Currently, 60 mg of ascorbate are recommended for CKD patients. Ascorbate’s role in the alteration of arterial blood pressure remains unclear, but anemic patients with functional iron deficiency might benefit from low-dose and short-term ascorbate supplementation [132].

Migliori et al. demonstrated that white wine and EVOO exert anti-inflammatory effects in both healthy subjects and CKD patients. This effect may be considered beneficial as they slow the CKD progression and prevent CVDs [125]. In 2020 and 2021, our research group carried out studies evaluating the potential beneficial effects of the EVOO minor polar compounds (MPCs) in CKD patients. It has been shown that the daily assumption of EVOO with a high MPCs content (at dose of 40 mL/day) seems to exert an important anti-inflammatory and antioxidant action in nephropathic patients. In particular, we examined two different types of Italian EVOO, demonstrating their positive effects on lipidic and purine metabolisms with a consequent slowing of CKD progression and CV protection [21,22].

Moreover, resveratrol (RESV), a polyphenol found especially in red wine, exerts protective effects against acute and chronic kidney injury through various mechanisms. In particular, RESV stimulates the action of Sirtuin 1 (SIRT1), namely through the activation of 5′ AMP-activated protein kinase (AMPK) via the inhibition of phosphodiesterase-4 (PDE-4) and the increase in cyclic adenosine monophosphate (cAMP), the downregulation of p53 by small interfering RNA. SIRT1 consequently inhibits the transforming growth factor β1 (TGF-β1) signaling, attenuates renal injury and ameliorates mitochondrial biogenesis. Furthermore, RESV regulates the gene expression and may control cell survival and/or apoptosis through global modulation of gene expression via deacetylation of transcription factors. RESV has been shown to protect the kidney of diabetic rats from OS induced by enhanced expression of fibronectin and collagen IV. RESV significantly alters the NO response and increases the endothelium-dependent vasorelaxation [133,134].

Another possible NBC associated with the improvement of endothelial function is vitamin D. Low serum concentration of this vitamin induces a decrease brachial artery flow-mediated dilation (FMD) and an increase of the soluble VCAM-1 (sVCAM-1) and the soluble endothelial leukocyte adhesion molecule-1 (sE-selectin) in CKD patients. It has been shown that the native vitamin D treatment improves the endothelium-mediated vascular responses in experimental studies [126]. 

The supplementation with cholecalciferol in CKD patients has been associated with a better calcium-phosphorus metabolism and consequently with a CV risk reduction [127]. In the literature, some studies demonstrated an improvement in endothelial function after cholecalciferol supplementation, but other studies did not show the same effect. Furthermore, in the latter studies vitamin D supplementation was used at a low dosage. A study conducted on non-diabetic CKD patients (stage III-IV according kidney disease improving global outcomes-K-DIGO guidelines [135]) evaluated the effects of the assumption of 300,000 IU of cholecalciferol at baseline and after eight weeks, showing a better vasomotor and secretory endothelial function in absence of side effects on the concentration of serum calcium and fibroblast growth factors (FGF)-23. Moreover, the authors did not report a worsening on the arterial stiffness [128]. HD patients, due to a secondary hyperparathyroidism, show a lower level of serum 25-hydroxyvitamin D3 (25-OH-D3) compared to healthy subjects. Another study evaluated the effects of cholecalciferol supplementation at a dose of 50,000 IU *per* week for eight weeks on endothelial function in HD patients. The authors highlighted increased FMD in percentage, suggesting an amelioration in CV risk [129]. Vitamin D status is associated with ED non only in HD patients but also in CKD patients under conservative therapy. In fact, another study revealed that cholecalciferol supplementation has beneficial effects on endothelial function in all pre-dialysis CKD patients. Therefore, it has been confirmed that 25-OH-D3 supplementation is associated with increased brachial artery FMD, also in CKD patients under conservative therapy. Moreover, these authors highlighted also decreased levels of sVCAM-1 and sE-selectin [136].

Vitamin D has also shown anti-inflammatory and anti-oxidative properties. It down-regulates the expression of renin and it has therefore acquired interest as a possible antihypertensive treatment in CKD patients. In vitro data support the concept of a direct effect of vitamin D on endothelial function, as it decreases the OS and it augments the levels of eNOS. Kendrick et al. conducted a double-blind randomized study on CKD adult patients with e-GFR comprised between 15–45 mL/min/1.73 m^2^. The study population was divided into two groups: one assumed cholecalciferol 2000 IU daily, and the second one assumed calcitriol 0.5 µg daily for six months. The authors did not detect any significant change in FMD in either group [130]. Even though the results about the role of vitamin D on ED are hard to generalize due to the small number of studies and the patients included, there were positive effects in both the fixed and the random model, indicating its benefits on endothelial function. The results also indicate that the highest impact has been described in younger patients, probably due to an earlier stage of ED, where vascular remodeling has not yet been established. These positive effects seem to be more evident with the cholecalciferol compared to calcitriol. This is possibly due to less increased calcium and phosphate levels with a vitamin D inactive form treatment [137]. 

The intake of oral food supplements and functional foods rich in NBCs and antioxidant molecules, such as EVOO, would seem to help the clinical management of CKD patients [21,22,118,138,139]. As reported, the mechanisms of action on ED and bioavailability of these natural metabolites in CKD patients are not yet investigated. These molecules are present in all plants, so it could be interesting to use these NBCs for the formulation of functional foods and oral food supplements in the treatment of the ED in CKD patients. Scientific research must investigate the role of metabolomics in these pathways starting from secondary metabolites with antioxidant and anti-inflammatory action tested in vivo and in vitro. Future clinical trials will be designed for the study of plasma antioxidant capacity and for high-performance liquid chromatography-diode array detector (HPLC-DAD) /MS analyses of secondary urinary and plasma metabolites in CKD patients, as we already performed for the EVOO MPCs [21,22,138].

Currently, a study registered with protocol number 223/20 by the Ethical Committee of Policlinico Tor Vergata, Rome (Italy), entitled “Evaluation of the possible energetic and beneficial action induced by the combination of an oral food supplement with adapted physical activity, on the onset and progression of uremic sarcopenia” is ongoing in the Tor Vergata, Foro Italico and Florence Universities (Italy). The study provides the administration of oral food supplements, characterized and standardized in NBCs content, evaluating their impact, alone or in combination with adapted physical activity, on uremic sarcopenia. The food supplements, at the best of our knowledge, for the first time, will allow the study of their biological and metabolomics activities in detail and in vivo.

## 6. Conclusions

**Figure 4:**
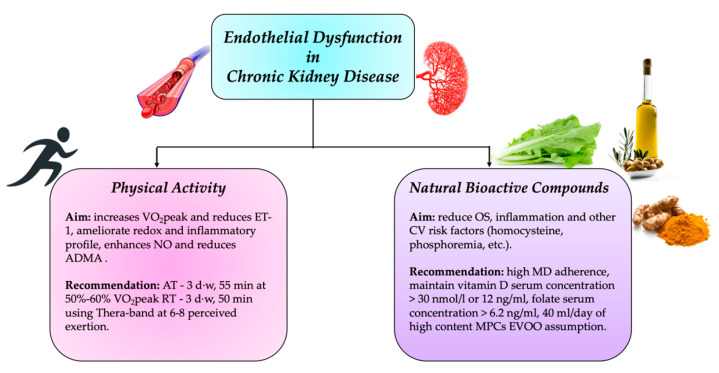
Physical activity and natural bioactive compounds recommended to counteract endothelial dysfunction in chronic kidney disease. ADMA, asymmetric dimethylarginine; d, day; CV, cardiovascular; EVOO, extra virgin olive oil; ET-1, endothelin 1; MD, Mediterranean diet; MPCs, minor polar compounds; NO, nitric oxide; OS, oxidative stress; RT, resistance training; VO2peak, peak oxygen uptake; w, week.

CKD, through various mechanisms, induces the development of ED, which participates in enhancing CV morbidity and mortality. The use of a treatment, free from side effects that does not impact on CKD progression and at the same time can improve ED, could represent a valid alternative therapy to reduce CV risk in CKD patients. Physical exercise and NBCs exert positive effects on these patients (Figure 4).

The available studies conducted on NBCs and on adapted physical activity examine few molecules, including only some subclasses already studied in the biomedical field, such as polyphenols. They have also investigated small sample size.

Therefore, it is necessary to carry out further clinical trials to evaluate and confirm effective role of NBCs and adapted physical activity in counteracting the ED in the course of CKD. Currently, the use of the traditional 2D cultures to study the endothelial cells of the different vascular beds would not seem to fully reflect both the functions and the gene expression profile of the different endothelial cells. Therefore, this method seems to be limiting. In this regard, kidney-on-a-chip techniques have been developed to better study and faithfully reproduce the environment in vivo [140,141]. Therefore, the future studies aimed at evaluating the cross-communication of kidney endothelial cells and the bioactivity of molecules would be represented by this technique.

Moreover, there is little evidence that the association of physical exercise and antioxidant supplementation may have an important role in the prevention or in the deterioration of CKD patients, but other studies are required to definitively prove their importance in this kind of patients.

## Figures and Tables

**Table 1:** Effects of physical activity on endothelial dysfunction in chronic kidney disease patients.

Author	Year	Study Population	Intervention	Outcome
Headley et al. [19]	2012	21 CKD patients, stage II–IV	AT (n = 10) 48 w, 3 d·w, 55 min at 50–60% VO_2_peak UC (n = 11)	AT increases VO_2_peak, reduces both resting and ambulatory HR, and increases LDL-Cholesterol
Headley et al. [109]	2014	46 CKD patients stage III, with diabetes mellitus and/or arterial hypertension	AT (n = 25) 16 w, 3 d·w, 15–30 min at 50–60% VO_2_peak UC (n = 21)	AT increases VO_2_peak and NOx; reduces ET-1, improves HRQOL no changes in aortic PWV.
Kirkman et al. [20]	2019	36 CKD patients under conservative therapy, stage III–V	AT (n = 19) 12 w, 3 d·w, 45 min at 60–85% heart rate reserve UC (n = 17)	AT improves VO_2_peak and microvascular function, maintains conduit artery endothelial function, no changes in arterial stiffness.
Van Craenenbroeck et al. [111]	2015	48 CKD patients under conservative therapy, stage III–IV	HAT (n = 19) 3 mons, 4 d·w, 10 min cycling at 90% of the HR achieved at the anaerobic threshold UC (n = 21)	HAT improves VO_2_peak and HRQOL, no changes in vivo vascular function and cellular markers for vascular function.
Corrêa et al. [112]	2020	55 HD patients for at least 3 mon	RT (n = 30) 12 w, 3 d·w, 50 min using Thera-band at 6–8 perceived exertion (intradialytic exercise) UC (n = 25)	RT improves sleep quality, ameliorate redox and inflammatory profile, enhances NO and reduces ADMA.
Rus et al. [113]	2005	18 HD patients	RT (n = 18) 8 w, daily, handgrip training + intermittent compression of the upper arm veins	RT increases radial artery diameters and endothelium-dependent vasodilation.

Abbreviations: ADMA, asymmetric dimethylarginine; AT, Aerobic Training; CHD, Cardiovascular Disease; CKD, Chronic Kidney Disease; d, day; ET-1, endothelin 1; HAT, Home-Based Aerobic Training; HD, hemodialysis; HR, Heart Rate; HRQOL, Health Related Quality of Life; LDL, low-density lipoprotein; mon, month; NO, nitric oxide; PWV, pulse wave velocity; UC, Usual Care; UG, Control Group; VO_2_peak, peak oxygen uptake; w, week.

**Table 2:** Effects of NBCs on ED in CKD patients.

Author	Year	Study Population	Intervention	Outcome
Di Daniele, N. [63]	2014	40 CKD male patients (stage II–III)	14 days of IMD followed by 14 days of IMOD	IMD and IMOD statistically reduce Hcy levels and the effect appears to be influenced by MTHFR genotypes.
Bennett-Richards, K. [122]	2002	25 normotensive CKD children (no-inflammatory etiology)	Supplementation of 5 mg/m^2^ surface area of folic acid for two 8-week periods separated by an 8-week washout period	Folic acid supplementation increases serum folate levels and reduces Hcy levels compared to the placebo group.
Lai, S. [123]	2020	41 CKD patients in conservative therapy	6 consecutive months of LPD (0.6 g/kg/day) (I) plus 19 g/day inulin (II) without inulin (control group)	LPD associated with inulin supplementation improved lipid and glucose metabolism and reduced systemic inflammation.
Bennett-Richards, K. [124]	2002	21 normotensive CKD children (stage IV) with ED	4 weeks of L-arginine supplementation at 2.5 g/m^2^ surface area and 5 g/m^2^ surface area × 3/day, separated by a rest period of 4 weeks	The L-arginine supplementation does not appear to impact ED.
Migliori, M. [125]	2015	10 healthy subjects and 10 CKD patients (stage III–IV)	2 weeks of white wine (4 mL/kg body weight, volume 12%) and EVOO oil (ad libitum) assumption	Chronic inflammatory biomarkers were significantly reduced in CKD patients during the combined consumption of white wine and EVOO.
Romani, A. [21]	2020	27 CKD patients in conservative therapy	40 mL/day of EVOO assumption for 9 weeks: (n = 13) with medium MPC content (>400 mg/L) and (n = 14) with high MPC content (>700 mg/L)	Daily consumption of EVOO 40 mL with high content of MPC improves lipid and purine metabolism and kidney function.
Noce, A. [22]	2021	40 CKD patients in conservative therapy	40 mL/day of EVOO assumption for 9 weeks and evaluation after 2 months of wash-out period: (n = 20) with medium MPC content (>400 mg/L) and (n = 20) with high MPC content (>700 mg/L)	Daily EVOO assumption with high MPC seems to exert anti-inflammatory and antioxidant action in CKD patients, that persist even after the washout period.
Zhang, Q. [126]	2015	71 CKD patients in conservative therapy with low vitamin D serum level	Oral supplementation of 50,000 units of cholecalciferol once a week for 12 weeks	Vitamin D supplementation can improve ED in CKD patients in conservative therapy.
Capusa, C. [127]	2016	87 CKD patients (stage IIIb)	-	Hypovitaminosis D is associated with subclinical peripheral arterial disease, higher serum alkaline phosphatase level and higher abdominal aortic calcifications score.
Chitalia, N. [128]	2014	26 non-diabetic CKD patients (stage III–IV)	Oral supplementation of 300,000 units cholecalciferol at baseline and after 8-weeks	Vitamin D improves endothelial vasomotor and secretory function, without any significant effect on arterial stiffness, calcium and FGF-23 levels.
Karakas, Y. [129]	2017	29 CKD patients on dialysis and 20 healthy controls	Oral supplementation of 50,000 units cholecalciferol for 8 weeks	Vitamin D supplementation reduces ED, increases percent flow-mediated dilation and could prevent CVD in dialysis patients.
Kendrick, J. [130]	2017	128 CKD patients (stage IIIb-IV) with low serum vitamin D level	Oral supplementation of cholecalciferol (2000 units/day) or calcitriol (0.5 μg/day) for 6 months	Six months of calcitriol or cholecalciferol supplementation did not improve vascular endothelial function or improve inflammation in CKD patients.

Abbreviation: CKD, chronic kidney disease; CVD, cardiovascular disease; ED, endothelial disfunction; EVOO, extra virgin olive oil; FGF-23, fibroblast Growth Factor-23; Hcy, homocysteine; IMD, Italian Mediterranean diet; IMOD, Italian Mediterranean organic diet; LPD, low protein diet.

## Data Availability

Not applicable.

## Abbreviations

25-OH-D3	25-hydroxyvitamin D3
ADMA	Asymmetric dimethylarginine
AGEs	Advanced glycation end products
AMPK	AMP-activated protein kinase
apoB	Apolipoprotein B
BP	Blood pressure
BW	Body weight
cAMP	Cyclic adenosine monophosphate
CKD	Chronic kidney disease
CKD-MBD	CKD-mineral bone disorder
CRP	C-reactive protein
CV	Cardiovascular
CVDs	Cardiovascular disease
DDAH	Dimethylarginine dimethylaminohydrolase
DM	Diabetes mellitus
eNOS	Endothelial nitric oxide inhibitor sintetase
ED	Endothelial dysfunction
EFSA	European food safety authority
e-GFR	estimated- glomerular filtration rate
ER	Estrogenic receptor
ESRD	End-stage renal diseases
EVOO	Extra virgin olive oil
FGF	Fibroblast growth factors
FMD	Flow-mediated dilation
GFR	Glomerular filtration rate
Hcy	Homocysteine
HD	Hemodialysis
HPLC-DAD/MS	High-performance liquid chromatography-diode array detector
Hs-CRP	High sensitivity C-reactive protein
ICAM-1	Intracellular adhesion molecule-1
IGF-1	Insulin-like growth factor-1
IL	Interleukin
IMD	Italian mediterranean diet
IMOD	Italian mediterranean organic diet
iNOS	Nitric oxide synthase
IS	Indoxyl sulfate
KDIGO	Kidney disease improving global outcomes
LDL	Low-density lipoprotein
L-NMMA	NG-monomethyl-L-arginine
LPD	Low-protein diet
MCF	Macrophage chemotactic factor
MCP	Monocyte chemoattractant protein
MD	Mediterranean diet
MetS	Metabolic syndrome
MMPs	Matrix metalloproteases
MPC	Minor polar compounds
MTHFR	Methylenetetrahydrofolate reductase
NADPH	Nicotinamide adenine dinucleotide phosphate-oxidase
NBCs	Natural bioactive compounds
N-CAD	Neural cadherin
NO	Nitric oxide
NOS	Nitric oxide inhibitor sintetase
OS	Oxidative stress
PDE-4	Phosphodiesterase-4
PECAM-1	Platelet endothelial cell adhesion molecule-1
PWV	Pulse wave velocity
RESV	Resveratrol
ROS	Reactive oxygen species
RRT	Renal replacement therapy
RT	Resistance training
SBP	Systolic blood pressure
sE-selectin	Soluble endothelial leukocyte adhesion molecule-1
SIRT1	Sirtuin 1
sVCAM-1	Soluble vascular cell adhesion molecule-1
TGF- β1	Transforming growth factor β1
VCAM-1	Vascular cell adhesion molecule-1
VE-CAD	VE-cadherin
VO_2_max	Maximal oxygen uptake
VO_2_peak	Peak oxygen uptake
